# Special Issue: “Rational Design and Synthesis of Bioactive Molecules”

**DOI:** 10.3390/ijms25189927

**Published:** 2024-09-14

**Authors:** Irena Kostova

**Affiliations:** Department of Chemistry, Faculty of Pharmacy, Medical University, 2 Dunav St., 1000 Sofia, Bulgaria; irenakostova@yahoo.com

The rational design of novel bioactive molecules is a critical but challenging task in drug discovery [1,2,3,4]. The design of newly generated drugs with definite pharmacological action is a complicated and specific problem, and the used computational methods should benefit from the input of prior information and knowledge [5,6]. 

Over the last few decades, many thousands of compounds have been synthesized and each of them has been tested experimentally to see if it has the desired properties [7,8,9,10]. Only a few potential drugs would eventually prove successful. The collective investigations between biologists and chemists in the field of effective rational drug design and optimization have produced numerous advantageous target-oriented lead compounds for different pharmacological systems [11,12,13,14,15] and for many diseases, including cancer [16,17,18,19], viruses [20,21,22], immune response [23,24], and the overproduction of reactive oxygen radicals [25,26]. The drug discovery process has, thus, relied on the skills of synthetic organic and inorganic chemists to provide an unlimited supply of novel biologically active compounds for pharmacological screening. That is why there would be obvious benefits if the effects of a given molecule could be predicted and molecules could be designed to fulfil a specific purpose.

The computer-aided design and prediction of the bioactivities remain a thought-provoking research area since it is not possible to test all of the newly projected molecules and to find the most active among them. Despite the great potential activity of bioactive candidates, they often show essential limitations and shortcomings, including poor solubility or stability, interactions with macromolecules, dose-dependent adverse effects, and intrinsic and acquired resistance, which must be considered for improving their activity, specificity, selectivity, and bioavailability and for their possible therapeutic application. A limited understanding of the precise modes of action of many new drug candidates complicates optimization efforts and necessitates additional investigations to gain systematic information on the respective bioprocesses [27,28].

Current chemo-physical strategies and bioinformatic studies for the examination of target–molecule interactions and structure–activity relationships, as well as the role of chemistry-directed approaches on the design and optimization of bioactive compounds, have been widely discussed in the literature devoted to drug discovery and development. Therefore, predicting the biological activity of newly designed, synthesized, and screened compounds is an imperative challenge in drug research, and significant progress has been made in this area [29]. Integrated computation-based methods involving quantitative structure–activity relationship (QSAR) modelling, molecular dynamics, and molecular docking computational simulations have been extensively used in drug development research [30]. The computation-based technologies allow the association of the structural features of investigated molecules to their biological action, the prediction and estimation of their bioactivities and physicochemical characteristics, and the determination of the molecular interaction mechanisms. 

Consistent with the high standards of International Journal of Molecular Sciences, the Special Issue titled “Rational Design and Synthesis of Bioactive Molecules” consists of eight research papers, which cover a broad array of topics in current research on various aspects of rational drug design with up-to-date information and recent advances in this research area.

The 1,2,3-triazole heterocycle is an important pharmacophore in medicinal chemistry that has attracted consideration in recent years because its derivatives have exhibited a range of biological properties, such as antimicrobial [31,32], antineoplastic [33], antioxidant [34,35], and anti-inflammatory properties [36]. The broad pharmacological applications and attractive photochemical characteristics of the 1,2,3-triazole derivatives [37] have made them interesting objects in various scientific fields, including biology, medicine, chemistry, and physics. The rational utilization of 1,2,3-triazole-based derivatives represents an attractive approach in the development of new drug candidates satisfying the chemical strategies and biological outcomes [38]. Safronov et al. [39] reported the synthesis and optical properties of novel biologically active 2-aryl-1,2,3-triazole acids and their sodium salts. The physicochemical characterization of these compounds has been studied theoretically and experimentally. The authors have proven that the novel sodium 2-phenyl-5-(pyrrolidin-1-yl)-2*H*-1,2,3-triazole-4-carboxylates were effective water-soluble blue fluorophores with excellent photophysical properties, making them good candidates as selective sensors for the recognition and identification of enzymes and receptors in a biological environment. 

The molecular structures and detailed vibrational characterization of six 1,2,3-triazoles-based compounds with possible anticancer and antioxidant activity have been theoretically and experimentally studied by the same research group [40]. The triazoles’ specific behaviour in biosystems is connected with the existence of the N-atoms in their heterocycles, that, along with heteroatoms of different substituents, are able to bind the active sites of numerous enzymes and receptors, forming intermolecular bonds. 2-Aryl-1,2,3-Triazol-5-Carboxylic acids and their anion forms have been optimized by using accurate scaling procedures. The impact of different substituents on the optimized structures and atomic charge distribution has been investigated. The structural, electronic, and spectroscopic characterization and the obtained relationships of the studied compounds could be useful for the selection of appropriate substituents and for the further design of new derivatives with enhanced biological action. 

Flavonoids are a class of polyphenolic secondary metabolites with many health benefits [41], including antioxidant [42], anticancer [43,44], and anti-inflammatory [45] activities. One of the most thoroughly studied properties of this large family of polyphenolic compounds is their antioxidant activity. Olszowy-Tomczyk and Wianowska’s paper [46] has described the antioxidant properties of biologically active flavonoids (myricetin, quercetin, and kaempferol) and their binary mixtures. Polyphenolic compounds display various antioxidant effects involving the prevention of the formation of ROS, scavenging free radicals, producing coordination complexes with pro-oxidant metals, and repairing the damage caused by reactive species. Studying the antioxidant activity of such flavonoids in mixtures is of great interest based on their number and the complex nature of the observed antioxidant effects. The authors have discussed the antioxidant properties of naturally occurring flavonoid aglycones (myricetin, quercetin, and kaempferol) and their binary mixtures tested by ABTS^●+^ and DPPH^●^ methods. In mixtures, the dominant resulting effect has been found to be the antioxidant antagonism, which depended on the type of the components, concentrations, and the methods applied to evaluate their antioxidant properties. The observed effects of the mixtures were dependent on their interactions and the formation of intramolecular H-bonds between phenolic functional groups. These results could be valuable for the future rational design of similar compounds and control of functional food ingredients.

Zhang et al.’s paper [47] examined the influence of polymer blends on in vitro release and degradation as well as on the pharmacokinetic parameters of moxidectin-loaded PLGA microspheres. Four formulations of moxidectin-loaded microspheres have been prepared by means of the oil-in-water emulsion solvent evaporation method by blending high- and low-molecular-weight poly-(D, L-lactic-co-glycolic acids) (PLGAs) with various ratios. These formulations have been characterized for particle size, surface morphology, drug loading, and physical states. Additionally, the pharmacokinetics of moxidectin-loaded microspheres have been estimated. PLGA has been widely used for sustained-release drug delivery because of its good biocompatibility and biodegradable properties. PLGA microspheres exhibit a range of benefits over traditional drug delivery systems, including long-acting drug release duration, reduced dosing frequency, improved tolerance, low toxicity, and costs [48,49]. The authors have proven that the blending of low-molecular PLGAs in different ratios did not produce an alteration in the release mechanism of microspheres but accelerated the drug release of microspheres and radically reduced the lag phase. The obtained results would provide an adequate theoretical basis for the control of microspheres’ release in order to eliminate or remarkably reduce the lag phase of hydrophobic drug-loaded microspheres.

During the last pandemic, there have been significant advances in the discovery of efficient antiviral agents [50]. In spite of the intense progress of antiviral therapeutic strategies, no specific treatment for coronavirus disease COVID-19 is currently available. Severe acute respiratory syndrome coronavirus 2 (SARS-CoV-2) belongs to a large family of viruses called beta-coronaviruses. The effectiveness of vaccines against SARS-CoV-2 is threatened by the appearance of new viral variants. Development of new agents targeting the infection by inhibition of viral entry, replication, release, and virus-induced inflammation is urgently needed [51]. Heparan sulfate has been found to play a significant role in virus–cell receptor interactions, and its mimetics represent potential inhibitors of virus attachment and entry. Li et al. [52] have established a unique pentasaccharide library composed of 14,112 species covering the probable sulfate substitution on different sugar units of heparan sulfate. Molecular modelling has been carried out to estimate the possible antiviral effects of the tested pentasaccharides, and the lead compound GlcNS-GlcA-GlcNAc3S-IdoA-GlcNAc, known as AD08043, has been identified. The main computational techniques that drive structure-based drug design are molecular docking and molecular dynamics simulations. These theoretical, computational techniques have been performed to find out the fluctuation and conformational variations of AD08043 under physiological environments. The obtained results afford important information for the mechanism of action and the design of heparan sulfate mimetics as potential anti-SARS-CoV-2 therapeutic candidates. 

Another major threat to public health is the so-called antimicrobial resistance. The extensive use of antibiotics has led to the rapid development of numerous drug-resistant strains. The great rise in antibiotic-resistant bacteria demands the discovery of antibiotics with novel structures and completely new mechanisms of action. Habeeb Mohammad et al. [53] have designed a 37-member library of α-aminocyclobutanone amides and sulfonamides, tested for inhibition of the bacterial enzyme diaminopimelate desuccinylase (DapE), which is a key antibiotic target, and identified several inhibitors with micromolar inhibitory potency, based on molecular docking studies. DapE is an important enzyme involved in the biosynthesis of lysine. It is related to the enzyme acetylornithine deacetylase and other amidases and peptidases. DapE is recognized as an antimicrobial target; hence, agents that inhibit its catalytic activity are required. Cyclobutanones are known for their ability to act as peptide- and transition-state mimetics inhibiting different families of proteases. Molecular docking shows a potential use of these compounds in the identification of key groups in the interaction with DapE. This series of the studied compounds provides leads for further optimization of the structures of new DapE inhibitors.

Zhang et al. [54] have collected a dataset of 955 MIC values of pleuromutilin derivatives to construct a 2D quantitative structure–activity relationship (QSAR) model and a 3D-QSAR model. Based on the obtained results from the QSAR study, new antimicrobial pleuromutilin compounds with thiol-functionalized side chains have been designed, synthesized, and screened for their in vitro antibacterial activity against *Staphylococcus aureus* and Methicillin-resistant *Staphylococcus aureus*. The natural compound Pleuromutilin, produced by the *Pleurotus mutilus,* has a specific tricyclic diterpene structure. Modifications of its structure have led to many derivatives with improved antibacterial activity for veterinary and human applications. The developed QSAR models have shown good prediction accuracy and helped to improve the activity and to find new potent pleuromutilin derivatives.

Natural chalcones, occurring as secondary plant metabolites, have shown a wide range of biological activities described in the literature. Their derivatives, 4′-hydroxychalcones, however, have scarcely been explored. Biologically active 4′-dihydrochalcones have been isolated from many medicinal plants and from the resin called ‘dragon’s blood’. The common pathway for most microbial biocatalysts is the chemo-selective reduction of the α,β-unsaturated ketone moiety to dihydrochalcones. Chlipała et al. [55] have studied 4′-hydroxychalcone biotransformation processes and determined the potentials of using biocatalysis in deep eutectic solvents (DESs) to obtain 4′-dihydrochalcones as model compounds. These processes are facilitated by whole-cell biocatalysts such as bacteria, fungi, cyanobacteria, and, predominantly, yeast. The studied biotransformation reactions have been carried out in a culture of the yeast *Yarrowia lipolytica* KCh 71 and also in cultures of strains of the genera *Rhodotorula* and *Debaryomyces* with high oxidoreductase activity. Of the additional yeast strains studied, *Rhodotorula marina* KCh 77 and *Rhodotorula rubra* KCh 4 have also shown good biocatalytic activity for the bio-reduction processes. The used ‘green solvents’, DESs, are known as biodegradable, nonhazardous, cheap, and easy to prepare, and they have been progressively used in numerous processes of extraction, chemical synthesis, and biotransformation. Additionally, the usage of DESs can contribute to process efficiency as a result of the increased bioavailability of the substrate. This study highlighted the potency of yeasts as biocatalysts and the prospect of using DESs as a reaction medium for the bio-reduction of 4′-hydroxychalcones. 

The promising and rapid development of rational design, applied in the discovery of new lead drugs, is primarily attributed to the incredible progress in computer and statistical sciences, bioinformatics, structural biophysics, biological chemistry, nanotechnology, molecular enzymology, and pharmaceutical chemistry. Therefore, rational computer-aided drug design represents a multidisciplinary, stimulating, and progressively growing field of research. The application of the known experimental and theoretical knowledge aims to decrease the cost, time, and laboratory expenses in the research of potential drugs with new structural features to modify their binding properties and pharmacokinetic activity and to elucidate their structure–activity relationship. Despite the promising but sporadic results, methodological systematic studies on structure–action relationships and on possible cellular mechanisms of action of many prospective drug candidates are still not sufficient. 

Rational drug design approaches are continually improving, and a broader variety of drug targets are being recognized by these methods. Advances in molecular modelling, developments in catalyst design, biological activity assays, and experimental structure determination methods to provide wider information have an important impact on how rational drug design is performed. Enlarged structural diversity requires precise consideration of many additional factors that strongly impact the bioactive compounds binding affinity, e.g., solvation, entropy, intermolecular and intramolecular interaction energies, hydrophobic effects, and conformational flexibility. The variations in physicochemical properties can change the absorption, distribution, metabolism, excretion, and toxicity profile of the biomolecules. Additional efforts to strengthen this field of research should address some of these important issues. 

The Special Issue “Rational Design and Synthesis of Bioactive Molecules” provides current perspectives on this interdisciplinary and rapidly developing area of research, as evident in the wide variety of problems and scientific approaches addressed. This issue, providing important drug design ideas, may shed light on the future development of promising drug candidates and can afford some novel concepts and enthusiasm for further investigations of various prospective strategies for the rational design of bioactive compounds having an impact on medical practice. 
